# Integrated Assessment of Humoral and Cellular SARS-CoV-2 Immunity Using ELISA and a Gold Nanoparticle-Based IGRA

**DOI:** 10.3390/ijms27198664

**Published:** 2026-09-28

**Authors:** Aleksandra Todorović, Danica Ćujić, Zorica Knežević Jugović, Petar Uskoković, Dušica Stojanović, Marija Gnjatović

**Affiliations:** 1 Institute for the Application of Nuclear Energy INEP, University of Belgrade, Banatska 31b, 11080 Belgrade, Serbia; aleksandra.todorovic@inep.co.rs (A.T.); marijad@inep.co.rs (M.G.); 2 Faculty of Technology and Metallurgy, University of Belgrade, Karnegijeva 4, 11000 Belgrade, Serbia; zknez@tmf.bg.ac.rs (Z.K.J.); puskokovic@tmf.bg.ac.rs (P.U.); duca@tmf.bg.ac.rs (D.S.)

**Keywords:** SARS-CoV-2, AuNP conjugate, nano-IGRA, interferon-gamma release assay, T-cell immunity, humoral immunity, hybrid immunity, immune response correlation

## Abstract

Although SARS-CoV-2 immunity has been widely investigated, the relationship between antibody levels and cellular immune responses following different routes of exposure to SARS-CoV-2 remains not fully revealed. In this study, cellular immunity was assessed using a newly developed whole-blood interferon-gamma release assay (nano-IGRA) based on gold nanoparticle (AuNP)-conjugated SARS-CoV-2 antigens. AuNP-conjugated and soluble antigens were compared regarding interferon gamma (IFN-γ) responses and intra- and interassay reproducibility in 112 individuals with immunity acquired through infection, vaccination, or both. Humoral immunity was evaluated by enzyme-linked immunosorbent assay (ELISA). Overall, 88% of participants were seropositive. AuNP-conjugated Spike (S) and Nucleocapsid (N) SARS-CoV-2 antigens induced significantly higher IFN-γ responses than soluble antigens in all exposure groups (*p* < 0.001). AuNP conjugation reduced intraassay variability by 62.4% and 61.4% for S and N, respectively, and interassay variability by 71.7% and 68.3%, respectively. Antibody levels correlated significantly with IFN-γ responses to S (ρ = 0.358, *p* < 0.001) and N (ρ = 0.309, *p* < 0.001). An interesting finding is that cellular responses were also detected in some seronegative individuals. Nano-IGRA, based on a novel AuNP-based approach, provides antigen-specific cellular immune assessment, combining enhanced IFN-γ responses with improved reproducibility. Further studies should establish diagnostic cut-offs, sensitivity, specificity, and clinical performance.

## 1. Introduction

At the beginning of 2020, a novel virus identified as severe acute respiratory syndrome coronavirus-2 (SARS-CoV-2) caused COVID-19 disease. The disease was declared a pandemic by the World Health Organization (WHO), spread worldwide and has caused over 7.1 million deaths in the past few years [1]. The enormous burden on the health system, dramatic lifestyle changes and the economic crisis required solutions that could effectively put the pandemic under control and eradicate or prevent any potential one in the future.

SARS-CoV-2 virus elicits a strong humoral and cellular immune response [2,3], expected to provide long-term immunity, either after SARS-CoV-2 infection and/or vaccination. Assessment of SARS-CoV-2 immunity was an effective and useful tool for the development of novel vaccine strategies against this virus, monitoring the emergence of novel virus variants [3] and gaining better understanding of the immunopathology of COVID-19.

Both memory B- and T-cell responses against SARS-CoV-2 are developed within a few days of infection onset and last months after infection [2,3,4]. Although measurement of antibody titers is usual in viral infection, T cell immunity in COVID-19 attracted significant attention. This was particularly the case when there was no detectable seroconversion [5,6,7] or lower antibody titers were recorded in asymptomatic infection and mild disease [8,9], or in immunocompromised individuals or patients on immunosuppressive medications, in whom B cell immunity is hindered [10,11]. T-cell immunity against SARS-CoV-2 was documented in patients with multiple sclerosis and malignancies [5,6,12] and evaluated among healthcare professionals [13,14], in mild/asymptomatic/infection without seroconversion [7,8,15] and within age-specific groups [16,17] following COVID-19 infection and/or immunization. However, the relationship between SARS-CoV-2 -specific humoral and cellular responses across individuals with different routes of antigen exposure remains not fully characterized. Moreover, there is a lack of examination of the possible relationship between the levels of specific anti-SARS-CoV-2 antibody response and virus-specific T-cell response.

Within this study, both branches of the immune response in COVID-19 were evaluated using diagnostic tests: the antibody response was analyzed using an enzyme-linked immunosorbent assay (ELISA), SARS-CoV-2 IgG, based on RBD-S protein (developed and manufactured as an IVD medical device by INEP-Serbia), while the cellular response was examined using the in-house interferon-γ (IFN-γ) release immunoassay (IGRA), which is based on gold nanoparticles (AuNPs), nano-IGRA. Although the IGRA test has been proven to be a significant and practical diagnostic tool for the detection of COVID-19 cell-mediated immunity [6,18,19,20], the lack of reproducibility is still a limiting issue, even if IGRA tests are repeated under identical conditions [21,22,23,24]. The aim of this study was to improve the reproducibility of the IGRA test by the introduction of gold nanoparticles in the system. The role of nanoparticles is to bind, through their carboxyl, amino or sulfhydryl groups, to serum albumin (SA), the main plasma protein [25,26,27], which is extracted during sample processing in the IGRA method. Au-based nanosystems have been widely used for years now in different applications, such as biomedicine, drug delivery, photothermal therapy and diagnostics [28,29,30,31,32,33]. Conjugation of albumin with AuNPs can increase selective uptake, biocompatible functionalities, and biodistribution and affords the stabilization of systems [32,34,35,36]. These properties provide a rationale for investigating AuNP-conjugated viral antigens as an alternative to conventional stimulation with soluble antigens in IFN-γ release assays, potentially reducing variability and increasing the reliability of IGRA results when analyzing IFN-γ as a marker of cellular immunity. To the best of our knowledge, this study is the first case of an IGRA platform with incorporated gold nanomaterial (nano-IGRA) used to evaluate cellular immunity to SARS-CoV-2.

In this study, we developed and evaluated a novel AuNP-based whole-blood IGRA methodology (nano-IGRA) using AuNP-conjugated SARS-CoV-2 Spike (S) and Nucleocapsid (N) proteins for antigen-specific stimulation. The performance of the AuNP-conjugated antigens was directly compared with the corresponding soluble antigens by assessment of IFN-γ responses, as well as intraassay and interassay reproducibility. We subsequently applied the nano-IGRA methodology to 112 individuals with different SARS-CoV-2 exposure histories, including natural infection, vaccination, and combined infection and vaccination. In parallel, humoral immunity was assessed by ELISA, enabling evaluation of the relationship between antibody responses and cellular IFN-γ responses across different immune-status groups.

The primary objective of this study was to improve SARS-CoV-2-specific cellular immunity assessment by enhancing T-cell stimulation with AuNP-mediated antigen stimulation in order to achieve better signal response and reduce assay variation. Further, we used the nano-IGRA platform to investigate the complementary relationship between humoral and cellular immune responses following different routes of SARS-CoV-2 antigen exposure. The current study was designed as a cross-sectional study. Comprehensive clinical diagnostic validation, including determination of diagnostic cut-off values, sensitivity, specificity, and direct comparison with commercial IGRAs, remains for future studies.

## 2. Results

A total of 112 subjects were evaluated for SARS-CoV-2 immunity, following natural infection and/or vaccination (Figure 1). To assess the antibody and cell response to SARS-CoV-2 of each participant, 112 serum plasma samples were collected. According to the origin of immune response, participants were stratified into three groups: COVID-19 convalescents (*n* = 37), vaccinated (*n* = 35) and combined effect of COVID-19 and vaccination (*n* = 40).

The demographic and clinical characteristics of the study population are summarized in Table 1. Humoral immunity was tested with an ELISA assay, based on the obtained index values for IgG antibodies. Serostatus was defined, according to the IgG index values: seronegative or low, medium and highly seropositive. The categories of seronegative, low, medium, and highly seropositive were defined based on the distribution of IgG index values observed during extensive use of the assay in SARS-CoV-2 testing, with distinct ranges corresponding to different levels of serological response identified through systematic analysis of the measured values. The seronegative category comprised subjects who, one month after a positive SARS-CoV-2 PCR test or administration of the first vaccine dose, had an IgG antibody response below the established assay cut-off. Cellular immunity was analyzed with the interferon gamma release assay based on gold nanoparticles (nano-IGRA), where the values of IFN-γ released by T cells, after stimulation with SARS-CoV-2 S and N protein, were measured and compared.

### 2.1. Humoral Immunity

Out of 112 subjects tested, 99 participants (88%) had measurable specific SARS-CoV-2 IgG antibodies (seropositive), while 13 samples (12%) were negative (seronegative). Seropositive or seronegative antibody results (IgG index) are summarized in Table 2. Serostatus was defined by the value of the IgG index: seronegative or low, medium or highly seropositive. In the group of patients with COVID-19 infection, 40% (*n* = 15) were low seropositive, 24% (*n* = 9) medium seropositive and 22% (*n* = 8) highly seropositive. Similar results were obtained in the vaccinated group: 40% (*n* = 14) were low seropositive, 26% (*n* = 9) medium, and 17% (*n* = 6) highly seropositive. In the group of subjects with hybrid immunity, the majority of participants had seropositive statuses: 42% (*n* = 17) were medium and 32% (*n* = 14) were highly seropositive, while 18% (*n* = 7) participants were low seropositive. Seronegative subjects made up 14% (*n* = 5) of the COVID-19 group (Table 2, Figure 2A), 17% (*n* = 6) of the vaccinated (Table 2, Figure 2B) and only 5% (*n* = 2) of the COVID-19 + vaccination group (Table 2, Figure 2C). The percentages of patients with positive serostatus in the COVID-19, vaccination and COVID-19 + vaccination groups were 86% (*n* = 32), 83% (*n* = 29) and 95% (*n* = 38), respectively (Figure 2). Analysis of seroprevalence revealed that in the group of participants with hybrid immunity (participants had infection and were vaccinated), there were almost twice as few seronegative and twice as many high seropositive serum samples as in the other groups. Summarizing the entire set of antibody results (Table 2), regardless of how the patient acquired immunity to SARS-CoV-2, one in three people was either low (32%) or medium seropositive (31%) and one in four was highly seropositive (25%).

### 2.2. Cellular Immunity

The cellular immunity in all 112 subjects was evaluated using the interferon-γ release assay formulated with gold nanoparticles (nano-IGRA). Two viral proteins, Spike (S protein) and Nucleocapsid (N protein), both shown to provoke T-cell immune response, were used as T-cell inductors. Activation of T-cells after stimulation with viral proteins was monitored by measuring IFN-γ concentration in plasma.

The analytical performance of the nano-IGRA methodology was evaluated by comparing whole-blood stimulation with AuNP-conjugated S and N antigens with parallel stimulation using the corresponding soluble antigens (Table 3). By measurement of IFN-γ concentration in stimulated plasma, the intra- and interassay variability of nano-IGRA was evaluated. In addition, it was also investigated whether AuNP conjugation affected the magnitude of produced IFN-γ.

For both S and N antigens, AuNP conjugation resulted in a consistent reduction in intraassay variability compared with the corresponding soluble-antigen stimulation. In the COVID-19 infection group, the intraassay CV decreased from 8.3% to 3.2% for S and from 7.4% to 3.6% for N. A similar pattern was observed in vaccinated individuals, with CVs decreasing from 7.3% to 2.8% for S and from 7.7% to 2.7% for N. In the hybrid-immunity group, intraassay CV decreased from 6.9% to 2.5% for S and from 7.0% to 2.2% for N.

The same pattern was observed for interassay reproducibility. In the infection group, interassay CV decreased from 10.5% to 3.5% for S and from 10.1% to 2.9% for N. In vaccinated individuals, the corresponding CVs decreased from 15.0% to 3.6% for S and from 15.3% to 4.7% for N, while in the hybrid-immunity group they decreased from 12.8% to 3.8% for S and from 10.2% to 3.7% for N. In parallel with the reproducibility improvement, AuNP conjugation resulted in higher IFN-γ concentrations compared to the corresponding soluble-antigen stimulation for both S and N antigens across all three study groups. Therefore, the implementation of AuNPs in the IGRA test improved method sensitivity as well.

When the three study groups were considered together, the magnitude of the improvement in assay reproducibility became even more evident (Table 4). AuNP conjugation reduced the mean intraassay CV by 62.4% for S and 61.4% for N, while the mean interassay CV was reduced by 71.7% and 68.3%, respectively. Thus, AuNP-mediated antigen stimulation evidently decreased IGRA test variation, consistently for both S and N antigens.

The fact that AuNP conjugation both enhanced IFN-γ following T-cell stimulation and obviously improved intra- and interassay reproducibility supported the advantage of the AuNP-conjugated antigen design within the nano-IGRA methodology compared to conventional soluble-antigen stimulation.

Having established the improved analytical AuNP-based stimulation format, we further applied the nano-IGRA methodology to evaluate T-cell response in all 112 study participants.

#### 2.2.1. COVID-19 Infection Group

T-cell stimulation with S protein showed a similar pattern of IFN-γ secretion as antibody response. The lowest INF-γ levels at 980 pg/mL [IQR: 413–1995] were measured in the seronegative (but SARS-CoV-2 PCR-positive) group, followed by 1380 pg/mL [IQR: 515–2170] in low seropositive patients, then 1460 pg/mL [IQR: 985–2293] in the medium seropositive group, while the highest values were observed in highly seropositive patients (1625 pg/mL [IQR: 1150–2198]) (Figure 3A). There was no significant difference in S protein-induced T-cell response between participants with different serostatuses (seronegative vs. low seropositive, *p* = 0.607; low vs. medium seropositive, *p* = 0.713; medium vs. highly seropositive, *p* = 0.55; seronegative vs. highly seropositive, *p* = 0.278). The median IFN-γ response after exposure to N protein reached its highest value (2128 pg/mL [IQR: 1713–3081]) in subjects with the highest antibody levels (IgG index ≥ 80). Seronegative patients showed an intensity of N protein-induced T-cell response similar to low or medium seropositive patients (1710 [IQR: 783–2823] pg/mL, 1680 [IQR: 790–2530], and 1950 [IQR: 1630–2903] pg/mL, respectively) (Figure 3B). Stimulation with N antigen showed that there was no difference in the median IFN-γ level between patients with different antibody titers (seronegative vs. low seropositive, *p* = 0.784; low vs. medium seropositive, *p* = 0.155; medium vs. highly seropositive, *p* = 0.787; seronegative vs. highly seropositive, *p* = 0.43).

#### 2.2.2. Vaccination Group

Vaccinated participants without previous COVID-19 disease showed a similar profile of S protein-stimulated cellular immunity to patients infected with the SARS-CoV-2 virus. The measured median IFN-γ levels were: 940 pg/mL [IQR: 628–1645] in seronegative subjects, 1322 pg/mL [IQR: 678–2329] in low seropositive subjects, 1970 pg/mL [IQR: 892–2712] in medium seropositive subjects and 2528 pg/mL [IQR: 1686–2860] in highly seropositive subjects (Figure 4A). There was a significant difference in S protein-induced T-cell response between seronegative and highly seropositive subjects (*p* = 0.007), while there were no observed differences between other serostatus subgroups (seronegative vs. low seropositive, *p* = 0.174; low vs. medium seropositive, *p* = 0.346; medium vs. highly seropositive, *p* = 0.257). The median IFN-γ response after stimulation with N protein reached its lowest value (965 pg/mL [IQR: 722–1179]) in seronegative subjects, while in low, medium and highly seropositive subjects its value was 1570 pg/mL [IQR: 899–2111], 1455 pg/mL [IQR: 978–2230] and 1698 pg/mL [IQR: 789–2561], respectively (Figure 4B). The lowest median IFN-γ value was observed in seronegative participants. A significant difference was observed between seronegative and low seropositive participants (*p* = 0.0083), whereas no significant differences were observed between low and medium seropositive participants (*p* = 0.323) or between medium and highly seropositive participants (*p* = 0.457).

#### 2.2.3. COVID-19 Infection + Vaccination Group

The median IFN-γ secretion after exposition to S protein reached its lowest value (1242 pg/mL [IQR: 1020–1465]) in seronegative subjects and its highest value (2120 pg/mL [IQR: 1709–2622]) in highly seropositive subjects (Figure 5A). Patients with low and medium seropositivity had a similar level of median IFN-γ: 1535 pg/mL [IQR: 1150–1710] and 1490 pg/mL [IQR: 740–2255], respectively. A significant difference was observed in S protein-induced T-cell response between seronegative and highly seropositive subgroups (*p* = 0.043) and also between medium and highly seropositive subgroups (*p* = 0.046). That was not the situation with other groups (seronegative vs. low seropositive, *p* = 0.494, and low vs. medium seropositive, *p* = 0.66). Stimulation with N protein showed a cellular immunity with an increasing distribution of median IFN-γ values for antibody response: the minimal value (1325 pg/mL [IQR: 1170–1480]) was observed in seronegative patients, followed by 1700 pg/mL [IQR: 1250–2375] in low seropositive patients, then 2240 pg/mL [IQR: 1735–2880] in medium seropositive patients, while the maximum value was obtained in highly seropositive patients (2368 pg/mL [IQR: 1706–3171]) (Figure 5B). T-cell response provoked by N antigen showed a statistically significant difference in the median IFN-γ level between seronegative and highly seropositive patients (*p* = 0.004), as well as between low and medium seropositive subjects (*p* = 0.034). No difference was observed in seronegative vs. low seropositive (*p* = 0.25) or medium vs. highly seropositive patients (*p* = 0.83).

#### 2.2.4. Comparison of T-Cell Response of All Three Groups: COVID-19 Infection, Vaccination and COVID-19 Infection + Vaccination

To further evaluate the connection between humoral and cellular SARS-CoV-2 immunity, the values of the IFN-γ levels of patients with the same serostatus (seronegative/low seropositive/medium seropositive/highly seropositive) from all three groups were compared. In this comprehensive analysis, IFN-γ titers were expressed as geometric means with 95% confidence intervals (CIs). The IFN-γ response was detected despite the absence of a humoral response (seronegative) in 11 patients (Figure 6A). Interestingly, in the category of seronegative subjects, stronger reactivity of cellular immunity was shown in the group of patients who had natural infection with COVID-19 (1159 [95% CI: 242–2076] pg/mL for S protein and 1784 [95% CI: 855–2713] pg/mL for N protein) than in the group of vaccine recipients (1062 [95% CI: 647–1477] pg/mL for S and 934 [95% CI: 721–1147] pg/mL) or in the group of subjects who had COVID-19 and who were vaccinated (1243 [95% CI: 807–1679] pg/mL for S and 1325 [95% CI: 1021–1629] pg/mL for N). Patients with a low positive serostatus had a fairly uniform T-cell response (Figure 6B). Overall, IFN-γ levels after T-cell stimulation with S protein in the COVID-19 infection, vaccination and COVID-19 + vaccination groups were: 1445 [95% CI: 966–1924], 1632 [95% CI: 1161–2103] pg/mL and 1496 [95% CI: 1067–1925] respectively, that is, 1796 [95% CI: 1373–2219] pg/mL, 1479 [95% CI: 1068–1890] and 1879 [95% CI: 1103–2655] pg/mL, respectively, following exposition to N protein.

In subjects who developed solid antibody levels (medium seropositivity), the highest concentration of released IFN-γ was induced by the combination of COVID-19 infection and vaccination (1604 [95% CI: 1190–2018] pg/mL for S-induced and 2291 [95% CI: 1962–2620] pg/mL for N-induced T cell response), while lower concentrations were observed in patients who only had infection (1517 [95% CI: 1061–1973] pg/mL for S and 2124 [95% CI: 1618–2630] pg/mL for N) or were only vaccinated (1809 [95% CI: 1189–2429] pg/mL for S and 153 [95% CI: 1047–2059] pg/mL for N) (Figure 6C). The situation was similar in patients with the highest titers of antibodies (highly seropositive): the strongest SARS-CoV-2 T-cell response was obtained by the combined effect of COVID-19 disease and vaccination (2211 [95% CI: 1834–2588] pg/mL for S protein and 2471 [95% CI: 2063–2879] pg/mL for N protein), while the reaction was somewhat weaker in the group where cellular immunity was provoked only through vaccination (2365 [95% CI: 1757–2973] pg/mL for S and 1684 [95% CI: 944–2424] pg/mL for N) or infection (1793 [95% CI: 1255–2311] pg/mL for S and 2253 [95% CI: 1670–2836] pg/mL for N) (Figure 6D). No statistically significant difference was observed in any category of subjects with the same serostatus when the SARS-CoV-2 IGRA reactivity of all three groups was compared (*p* > 0.05).

#### 2.2.5. Correlation Between Humoral and Cellular Immune Responses

To further investigate the relationship between humoral and cellular immunity, Spearman correlation analysis was performed between SARS-CoV-2-specific IgG index values and IFN-γ responses induced by S and N antigens. Correlations were assessed separately in the COVID-19 infection, vaccination, and hybrid-immunity groups. The correlation coefficients and corresponding *p*-values are summarized in Table 5.

Spearman correlation analysis demonstrated a positive association between humoral and cellular immune responses in vaccinated and hybrid-immunity participants. In the vaccination group, IgG index values were significantly correlated with S-induced IFN-γ responses (ρ = 0.475, *p* = 0.004), whereas no significant correlation was observed with N-induced IFN-γ responses (ρ = 0.085, *p* = 0.626). In the hybrid-immunity group, significant positive correlations were observed between IgG index and both S-induced (ρ = 0.412, *p* = 0.0083) and N-induced IFN-γ responses (ρ = 0.371, *p* = 0.0185). When all participants were analyzed together, significant positive correlations were observed for both S (ρ = 0.358, *p* < 0.001) and N (ρ = 0.309, *p* < 0.001). Overall, these findings indicate that higher SARS-CoV-2-specific antibody levels were associated with stronger cellular IFN-γ responses, particularly following vaccination and hybrid exposure.

## 3. Discussion

Recently, a newly identified SARS-CoV-2 virus has managed to circumvent many of the adopted rules of immunology. The diversity of the immune response, the appearance of hypersensation of T-cells until their exhaustion and impairment [37], unusual multisystem manifestations of COVID-19 [38,39,40] and worldwide spreading required the united work of doctors and scientists in characterizing the immunopathogenesis of SARS-CoV-2 infection. The experience and knowledge gained during the COVID-19 pandemic and subsequent research regarding the relationship between the virus and host immunity will contribute to the readiness to fight against any potential pandemic in the future. In this report, the relationship between humoral and cellular immune response to SARS-CoV-2 was evaluated in 112 subjects, classified in three subgroups, based on immune response provocation. Within the first subgroup were patients who developed immunity after natural infection with COVID-19, in the second subgroup were vaccinated individuals, while in the third group were those with hybrid immunity, i.e., those who overcome COVID-19 infection and were vaccinated. Immune response was assessed based on indirect measurement of the level of produced SARS-CoV-2 -specific antibodies and the reactivity of the T-cell response by measuring IFN-γ in plasma.

While serology is a routine diagnostic procedure for the assessment of specific antibody levels, T-cell monitoring is not so frequent an approach. IGRA testing is commonly used in diagnosis of tuberculosis, particularly in latent and asymptomatic cases, as well as in monitoring of CMV infection in immunocompromised patients [41,42,43,44]. While serological assays are easy to perform in routine laboratories and could be fully automated, measurement of cellular immunity is considered to be a more complex operation. As it relies mostly on manual work, reproducibility is one of the main concerns with the interferon-γ (IFN-γ) release immunoassay (IGRA). The aim of the present study was to optimize IGRA testing by the introduction of gold nanoparticles (AuNPs): nano-IGRA. Recent studies have highlighted IGRA testing as a useful method to assess SARS-CoV-2 T-cell immunity [18,19,20,45], but it has also been observed that IFN-gamma values can vary during serial testing, despite consistent procedures [22,23,24]. This drawback was overcome by conjugation of AuNPs with SARS-CoV-2 antigens, which are used to stimulate T-cells in whole-blood samples. A potential explanation may involve the interaction of AuNPs with proteins present during whole-blood processing: in the incubation of AuNP-S protein or AuNP-N protein with heparinized blood, viral proteins activate T-cells, while in parallel blood plasma is separated and plasma albumin binds to gold nanoparticles. This nanoplatform manifests interesting properties: stability and biocompatibility is increased [27,28,46], and the functionality of the protein conjugated to AuNPs is preserved [32] and promotes the recruitment of target proteins [34,47]. These are important features for the application of AuNP-SA in biomedicine, especially in disease diagnostics, as it is a nanosystem that provides results characterized by reliability and reproducibility. One such application of Au is our in-house nano-IGRA, which was used to examine the COVID-19 T-cell response in all participant subjects. Gold nanoparticles have been shown to improve the diagnostic performance of the IGRA test for latent tuberculosis infection [48], but to the best of our knowledge this is the first time that such a platform has been applied to assess cellular immunity to SARS-CoV-2.

The present study provides a description of a novel methodological approach for the assessment of SARS-CoV-2-specific cellular immunity by combining AuNP-mediated antigen stimulation with a whole-blood IFN-γ release assay (Table 6). The novelty of the proposed nano-IGRA system resides in the integration of AuNP-mediated antigen presentation into the conventional whole-blood IGRA format. The AuNP-mediated antigen presentation strategy preserves the operational simplicity of whole-blood stimulation while introducing a nanoscale antigen presentation platform that can enhance antigen–immune cell interactions in vitro. In our study, this concept was demonstrated experimentally by the higher IFN-γ readout and significantly lower intra- and interassay variability observed with AuNP-conjugated antigens compared to their soluble counterparts (Table 3). The rationale for developing the proposed nano-IGRA system was driven by both practical diagnostic considerations and our routine experience during the COVID-19 pandemic. Although nanomaterial-based approaches have previously been explored in IGRA- or cytokine-release assay formats for other infectious diseases, to the best of our knowledge the integration of gold nanoparticle-conjugated SARS-CoV-2 antigens into a whole-blood IGRA format has not been previously reported. During the pandemic, humoral immunity was primarily monitored, and in some individuals unexpectedly low or undetectable antibody levels were observed despite prior SARS-CoV-2 exposure or vaccination. This highlighted the potential value of assessing cellular immunity. Interpretation of IGRA results can also be challenging when IFN-γ responses are close to the decision threshold or fall within a borderline/grey zone. Even commercial in vitro diagnostic tests may occasionally yield unclear or indeterminate results due to technical factors or interindividual variation in immune responses, requiring repeat testing. These considerations motivated us to explore whether incorporation of gold nanoparticles into the antigen-stimulation system could increase IFN-γ release by stimulated T-cells in order to improve IGRA test performance and result reliability. A resulting IFN-γ response upon AuNP-conjugated antigen stimulation indeed produced a stronger signal and improved assay reproducibility. However, formal assessment of diagnostic sensitivity, specificity, and gray-zone performance would require dedicated clinical validation.

Rather than evaluating antibody and T-cell responses as independent measures, we applied the developed nano-IGRA methodology together with ELISA-based antibody assessment in 112 individuals with different SARS-CoV-2 exposure histories, including natural infection, vaccination, and combined infection and vaccination. The study therefore addressed two complementary aspects: first, the analytical performance of AuNP-conjugated antigen stimulation compared with conventional soluble-antigen stimulation; and second, the applicability of this approach to the integrated assessment of humoral and cellular immune responses.

A major finding of the present study was the marked difference between soluble and AuNP-conjugated antigen stimulation in terms of improved reproducibility and sensitivity (Table 3 and Table 4). Both AuNP-S and AuNP-N induced significantly higher IFN-γ responses than their corresponding soluble antigens across all three study groups (Table 3). Importantly, this increase in secreted IFN-γ was accompanied by a substantial reduction in assay variability. For both S and N antigens, intra- and interassay CVs were consistently lower upon AuNP conjugation across all three study subgroups. When the three groups were considered together, intraassay variability was reduced from approximately 7.5% to 2.8% for S and from approximately 7.3% to 2.8% for N, while interassay variability decreased from approximately 12.8% to 3.6% for S and from approximately 11.9% to 3.8% for N (Table 4). Thus, the effect of AuNP conjugation on viral antigens was not just restricted to an increase in IFN-γ secretion, but also improved assay precision.

The results demonstrated that, overall, 88% patients were seropositive for SARS-CoV-2 IgG antibodies, while 12% were seronegative, regardless of how they acquired COVID-19 immunity. In addition, the combined effect of SARS-CoV-2 infection and vaccination creates a much stronger humoral response than the effect of vaccination or infection alone, and these findings are consistent with recent studies [13,57,58,59]. Our results indicate fairly similar distributions of antibody titers induced by COVID-19 disease and vaccination, and they correspond well with data recently reported [60]. Within our study cohort, regardless of how humoral immunity to SARS-CoV-2 was provoked, one in three participants was either low or medium seropositive and one in four was highly seropositive.

Infections caused by viruses, as well as vaccines, induce cellular immunity, which is an important link and represents an indicator of protective COVID-19 immunity [8,61,62]. Therefore, data on SARS-CoV-2 T-cell response are important [63]. We assessed the T-cell response using nano-IGRA: the concentration of IFN-γ released after antigen-specific T cell stimulation with S or N protein was measured. Our data demonstrated that S protein-induced or N protein-induced T cell immunity displayed IFN-γ secretion with a similar increasing distribution of values to antibody response: smaller values were obtained in patients with lower antibody levels, while higher values were observed in subjects with higher antibody titers. In all three study subgroups, a specific SARS-CoV-2 cellular response in seronegative hosts was detected. This is in agreement with some studies which have already indicated the presence of T-cell responses in the absence of humoral immunity [13,16,64]. One possible explanation of these finding is that a person who has been exposed to SARS-CoV-2 virus proteins does not necessarily synthesize antibodies. Exposure to very low concentrations of SARS-CoV-2 does not always trigger seroconversion, as T-cells can clear subclinical infection before B-cell activation [13,65]. Analyzing each group separately, we found that, in general, there were no significant differences between SARS-CoV-2 S-induced and N-induced T-cell response in seronegative, low seropositive, medium seropositive and highly seropositive patients.

To investigate a potential interdependence between humoral and cellular SARS-CoV-2 immunity, we compared the IFN-γ concentrations of patients with the same serostatus (seronegative/low seropositive/medium seropositive/highly seropositive) from all three subgroups. The combination of infection and vaccination induced the strongest T-cell immunity (hybrid immunity) in all categories with positive serostatus (low, intermediate and high seropositive patients), while in seronegative subjects the most dominant cellular response was developed by COVID-19 infection. Our data suggest that SARS-CoV-2 hybrid immunity is potentially the infrastructure of long-term protection, which is in agreement with recently published studies [66,67,68]. Notably, except in uninfected vaccine recipients, where S protein showed higher nano-IGRA reactivity than N protein (because the design of most vaccines is based on the S antigen), in all categories N protein elicited a stronger T-cell signal. These results correspond well with the suggestion by Zollner A. et al. that SARS-CoV-2 N protein is a potent trigger for T-cells [13]. Also, this is explained by the fact that the Nucleocapsid gene is characterized as highly conserved and stable [69]; therefore, the virus mutations that occur do not threaten the reliability of the diagnostic tool or future vaccines based on this protein.

This relationship was further examined by Spearman correlation analysis between IgG index values and antigen-induced IFN-γ responses. In the vaccination group, a significant positive correlation was observed between IgG index and S-induced IFN-γ response (ρ = 0.475, *p* = 0.004), whereas no significant correlation was observed between IgG index and N-induced IFN-γ response (ρ = 0.085, *p* = 0.626). In participants with hybrid immunity, significant positive correlations were observed between IgG index and both S-induced (ρ = 0.412, *p* = 0.0083) and N-induced IFN-γ responses (ρ = 0.371, *p* = 0.0185). In contrast, no statistically significant correlations were observed in the infection-only group for either S-induced (ρ = 0.239, *p* = 0.153) or N-induced IFN-γ responses (ρ = 0.215, *p* = 0.202). When all participants were analyzed together, significant positive correlations were observed between IgG index and both S-induced (ρ = 0.358, *p* < 0.001) and N-induced IFN-γ responses (ρ = 0.309, *p* < 0.001). These findings indicate that the relationship between humoral and cellular immunity was dependent on the mode of SARS-CoV-2 exposure. The strongest association was observed between IgG index and S-induced IFN-γ response in vaccinated individuals, which may reflect the common spike-antigen exposure provided by vaccination. In contrast, the hybrid-immunity group demonstrated significant associations for both S- and N-induced responses, consistent with the broader antigenic exposure resulting from the combination of infection and vaccination. The absence of statistically significant correlations in the infection-only group may reflect greater heterogeneity in immune responses following natural infection, including differences in viral exposure, disease severity, and the time elapsed since infection.

An important feature of the present study is that the nano-IGRA methodology was evaluated not only in a single clinical subgroup but across three distinct exposure profiles. The consistent reduction in intraassay and interassay CVs observed across these groups suggests that the improved analytical reproducibility of the AuNP-based stimulation was not limited to a particular immune-status category. This provides an important methodological foundation for the use of nano-IGRA in studies comparing cellular immune responses following different types of SARS-CoV-2 exposure.

The present study should nevertheless be considered a methodological and immunological evaluation rather than a complete clinical validation of a diagnostic IGRA. Diagnostic cut-off determination, sensitivity, and specificity were not established in the present study, and a direct head-to-head comparison with an established commercial SARS-CoV-2 IGRA was not performed. These analyses will be necessary to determine the diagnostic performance of the nano-IGRA platform and to establish whether the observed improvement in analytical reproducibility translates into improved clinical discrimination. In addition, larger independent cohorts and external validation will be required to assess the robustness of the methodology across different populations and pre-analytical conditions.

Beyond SARS-CoV-2, the modular nature of this platform may provide a basis for the development of innovative nanomaterial-based diagnostic approaches for the evaluation of cellular immune responses to other infectious diseases and, potentially, to allergen-specific immune responses. By adapting the antigen or allergen used for stimulation, AuNP-based antigen presentation could be further explored as a strategy for improving the analytical performance and reproducibility of whole-blood cellular immune assays.

## 4. Materials and Methods

### 4.1. Study Participants

In the period from August 2020 to March 2022, we analyzed a data set of 112 patients divided into three subgroups: (1) patients with confirmed COVID-19 infection (*n* = 37); (2) patients who were not infected but were vaccinated (*n* = 35); and (3) patients with hybrid immunity, i.e., those who had COVID-19 and who had been vaccinated (*n* = 40). Subjects included in this study who were only infected, as well as subjects who were vaccinated in addition to having COVID-19 infection, had a positive SARS-CoV-2 reverse transcription-PCR (RT-PCR) test result or a positive serologic determination (antibody/T-cell test) at some time point. The group of vaccinated subjects had negative PCR results, antibody titers below the cut-off value and no symptoms specific to COVID-19 prior to vaccination.

All participants signed a written informed consent and accepted their participation in this study approved by the Medical Ethical Committee of the Institute for the Application of Nuclear Energy (INEP, Belgrade, Serbia).

### 4.2. Detection of Humoral SARS-CoV-2 Immunity

Humoral response was analyzed using an enzyme-linked immunosorbent assay—ELISA (INEP, Serbia)—with detection of specific SARS-CoV-2 IgG antibodies against Spike (S) and receptor-binding domain (RBD) proteins. The positive and negative control samples were included in each assay run. A serum index with a cut-off value ≥ 20 was considered to be positive and is an indicator of the immune system’s response to a virus or vaccine. Based on the IgG index values obtained in the ELISA test, patients were divided into four groups: seronegative (index < 20), low seropositive (20 ≤ index < 40), medium seropositive (40 ≤ index < 80) and high seropositive subjects (index ≥ 80). This assay was performed on an ELISA reader (Wallac Multilabel Counter 1420; Perkin Elmer, Milano, Italy).

### 4.3. Detection of Cellular SARS-CoV-2 Immunity

Cellular SARS-CoV-2 immunity was assessed by in-house interferon-γ (IFN-γ) release immunoassay based on either soluble recombinant viral antigens or conjugation to nanomaterials (AuNPs). IGRA and nano-IGRA tests were performed in two phases: stimulation of T-cells in heparinized whole-blood samples with either S or N SARS-CoV-2 proteins and subsequent measurement of the concentration of released IFN-γ in separated plasma. Participants’ blood was collected in heparinized vacutainer tubes (Becton, Dickinson and Company, Franklin Lakes, NJ, USA) and stimulated with viral antigens, either in soluble form or as AuNP conjugates. Separated vials for stimulation (Figure 7) were customized and made in-house, with the introduction of colloidal gold nanoparticles (AuNPs). Stimulation was performed using two SARS-CoV-2 recombinant proteins (ACROBiosystems, Newark, DE, USA): Spike (S) and Nucleocapsid (N), selected to stimulate both CD4+ and CD8+ T-cells. Colloidal gold nanoparticles (DCNovations, Carlsbad, CA, USA) were conjugated with S or N protein at a concentration of 1 µg/mL according to the manufacturer’s instructions, and then stimulation vials were coated with 350 µL of this suspension. Participants’ heparinized blood samples were mixed for at least half an hour, then aliquoted at 1 mL in separate S or N protein vials. In addition, a 1 mL aliquot was poured into an empty vial in order to estimate basal IFN-γ secretion. The fourth aliquot was stimulated with 3.5 µg/mL of a non-specific mitogen, phytohemagglutinin (PHA; Sigma, St. Louis, MO, USA), as a positive control. After overnight stimulation at 37 °C, under a 5% CO_2_ atmosphere, plasma containing released IFN-γ was extracted. The levels of released IFN-γ were directly proportional to T-cell activation and measured using the Human IFN-g ELISA Set (BD Biosciences Pharmingen, San Jose, CA, USA) according to the manufacturer’s instructions.

### 4.4. Statistical Analysis

Continuous data are presented as medians and interquartile ranges, while categorical data are presented as numbers and percentages. Antibody levels (obtained from ELISA) and IFN-γ concentrations (obtained from nano-IGRA) are expressed either as medians with interquartile ranges (IQRs) or as geometric means with 95% confidence intervals (CIs). Continuous values and differences between groups were calculated by Student’s *t*-test or the Mann–Whitney test, when appropriate. Paired comparisons were performed using the Wilcoxon signed-rank test. Correlations between IgG index and IFN-γ responses were assessed using Spearman’s rank correlation coefficient. All statistical tests were two-tailed, and *p* < 0.05 was considered statistically significant. Statistical analysis was conducted using GraphPad Prism 8.3.1 (GraphPad Software Inc., San Diego, CA, USA).

## 5. Conclusions

In conclusion, the present study demonstrates a novel AuNP-based approach for antigen stimulation within a whole-blood IFN-γ release assay (nano-IGRA) for the assessment of SARS-CoV-2-specific cellular immunity. Compared with conventional soluble-antigen stimulation, AuNP-conjugated S and N antigens produced significantly higher IFN-γ responses while substantially reducing intraassay and interassay variability. The consistent improvement in assay reproducibility across infection, vaccination, and hybrid-immunity groups supports the methodological robustness of the AuNP-based stimulation approach.

Application of the nano-IGRA methodology to 112 individuals demonstrated that cellular immune responses provided complementary information to antibody measurements, including detectable IFN-γ responses in some seronegative individuals. Furthermore, Spearman correlation analysis demonstrated significant positive associations between IgG index and IFN-γ responses in vaccinated and hybrid-immunity participants, as well as when all participants were analyzed together, supporting the value of combined assessment of humoral and cellular immunity.

A potential limitation of the present study is the absence of a comparison nano-IGRA platform with a commercially available SARS-CoV-2 IGRA or an orthogonal T-cell assay. Furthermore, a full diagnostic validation study (including clinical sensitivity, specificity, and diagnostic cut-off determination) is needed and remains to be established in future studies performed on larger and appropriately characterized study cohorts.

Taken together, these findings support nano-IGRA as a promising platform for the methodological and immunological assessment of SARS-CoV-2-specific cellular immunity. Beyond SARS-CoV-2, the modular nature of this nanomaterial-based approach may provide a basis for developing innovative cellular immune-assessment platforms for other infectious diseases and, potentially, allergen-specific immune responses. By adapting the antigen or allergen used for stimulation, AuNP-based antigen presentation could be further explored as a strategy for improving the analytical performance and reproducibility of whole-blood cellular immune assays.

## Figures and Tables

**Figure 1:**
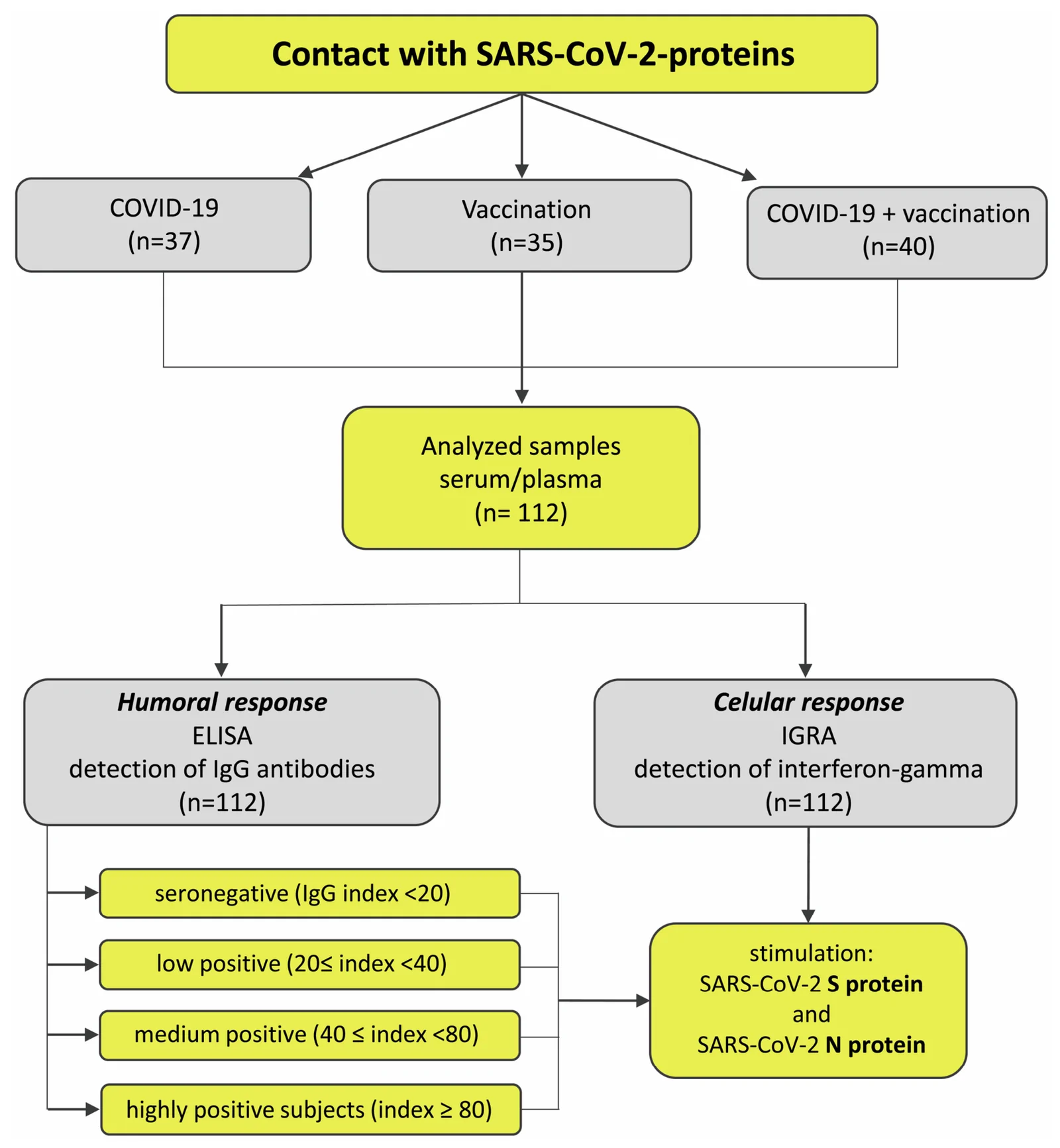
Flowchart representation of study design.

**Figure 2:**
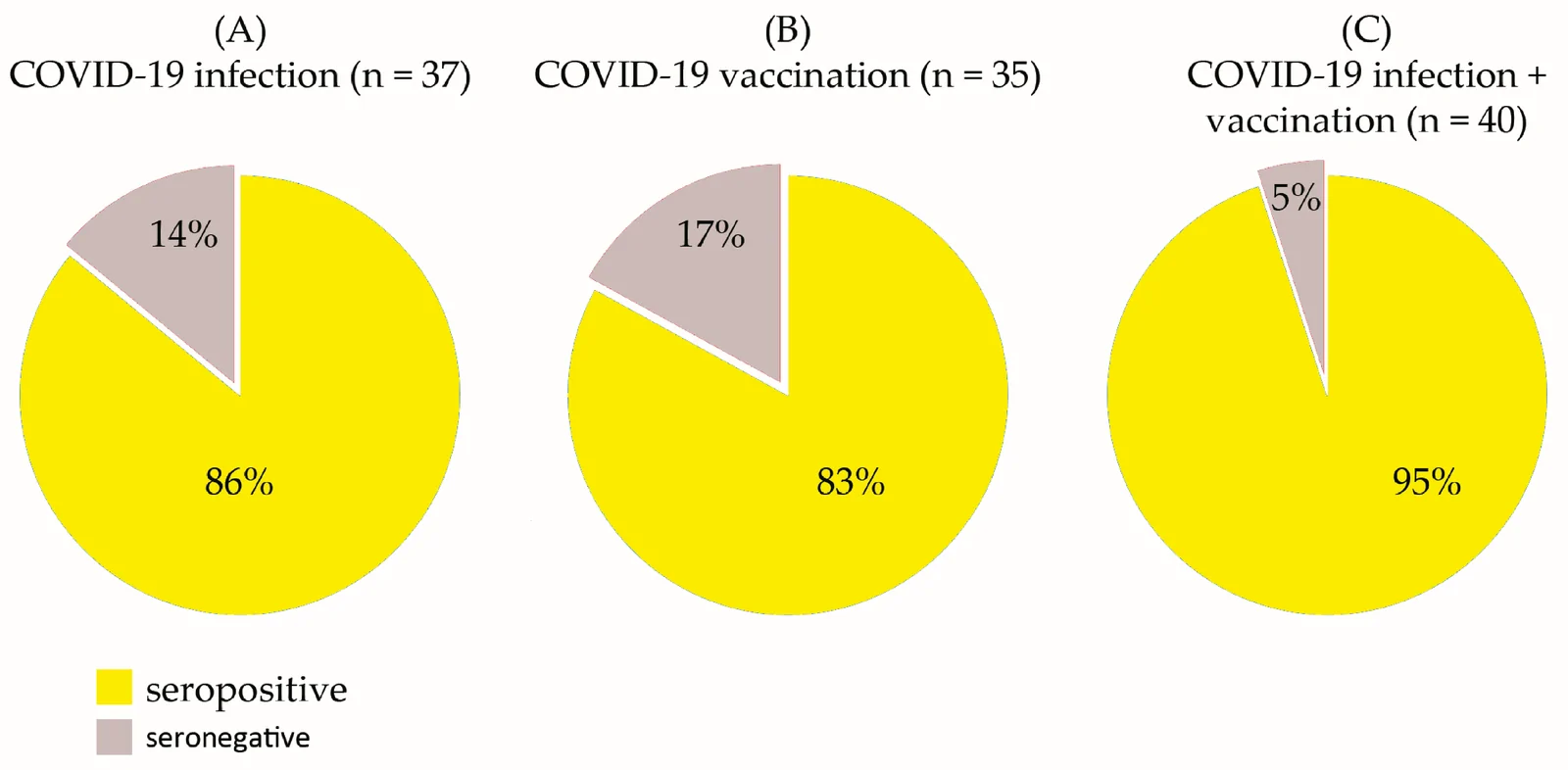
Specific SARS-CoV-2 IgG prevalence. The proportions of SARS-CoV-2 seropositive and seronegative subjects who were infected with COVID-19, subjects who were vaccinated with SARS-CoV-2 vaccines, and subjects who had COVID-19 and vaccination.

**Figure 3:**
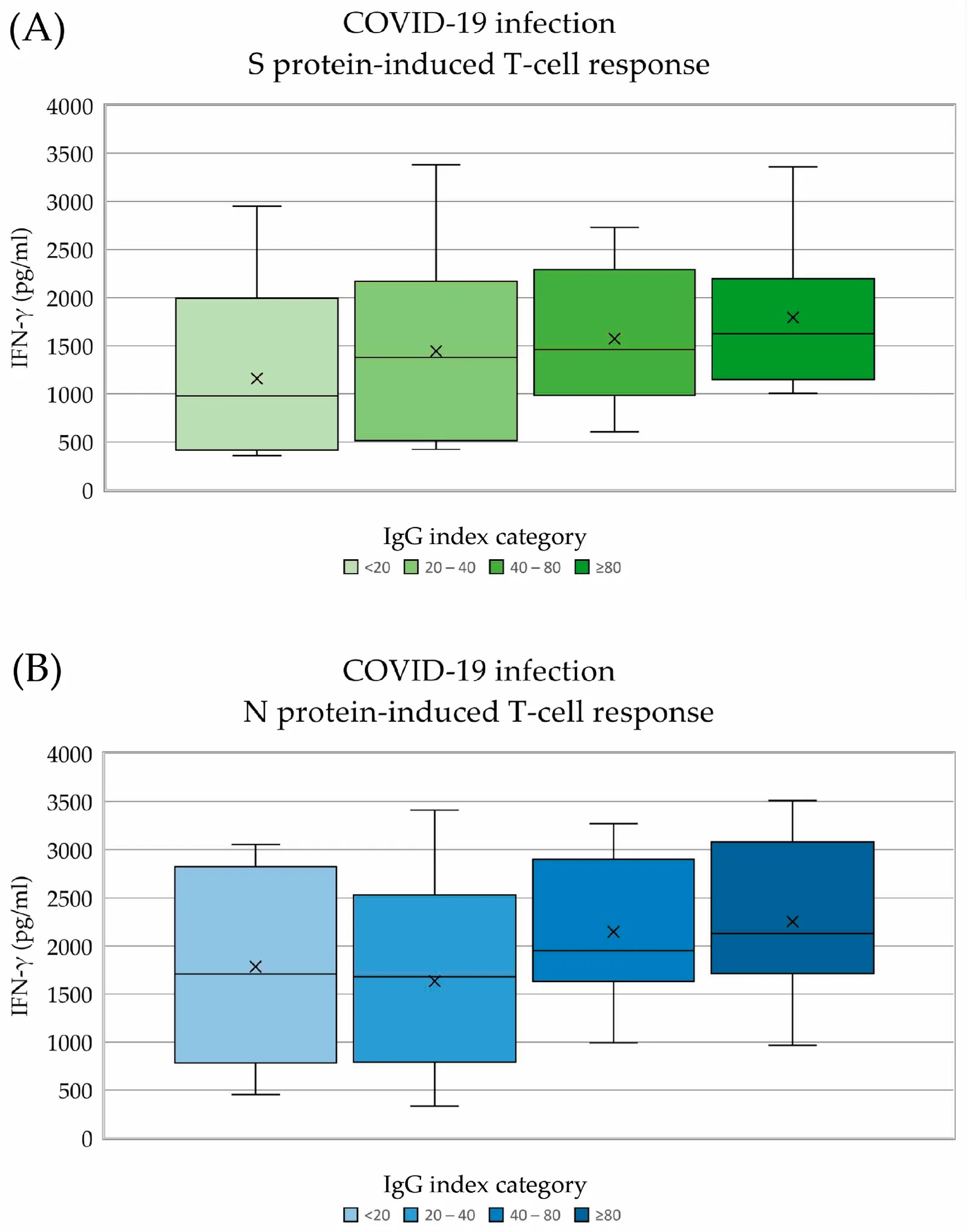
Interferon gamma (IFN-γ) release assay based on gold nanoparticles (nano-IGRA) for detection of cellular immune response to SARS-CoV-2 in COVID-19 convalescents, stratified in four subgroups according to IgG antibody levels (seronegative subgroup (IgG index < 20), *n* = 5; low seropositive group patients (IgG index 20–40), *n* = 15; medium seropositive subgroup (IgG index 40–80), *n* = 9; and highly seropositive subgroup (IgG index ≥ 80), *n* = 8). (**A**) IFN-γ response of whole blood stimulated with SARS-CoV-2 S protein. (**B**) IFN-γ response of whole blood stimulated with SARS-CoV-2 N protein. The upper whiskers extend from the hinge to the highest value that is within 1.5 × IQR of the hinge. The lower whiskers extend from the hinge to the lowest value within 1.5 × IQR of the hinge.

**Figure 4:**
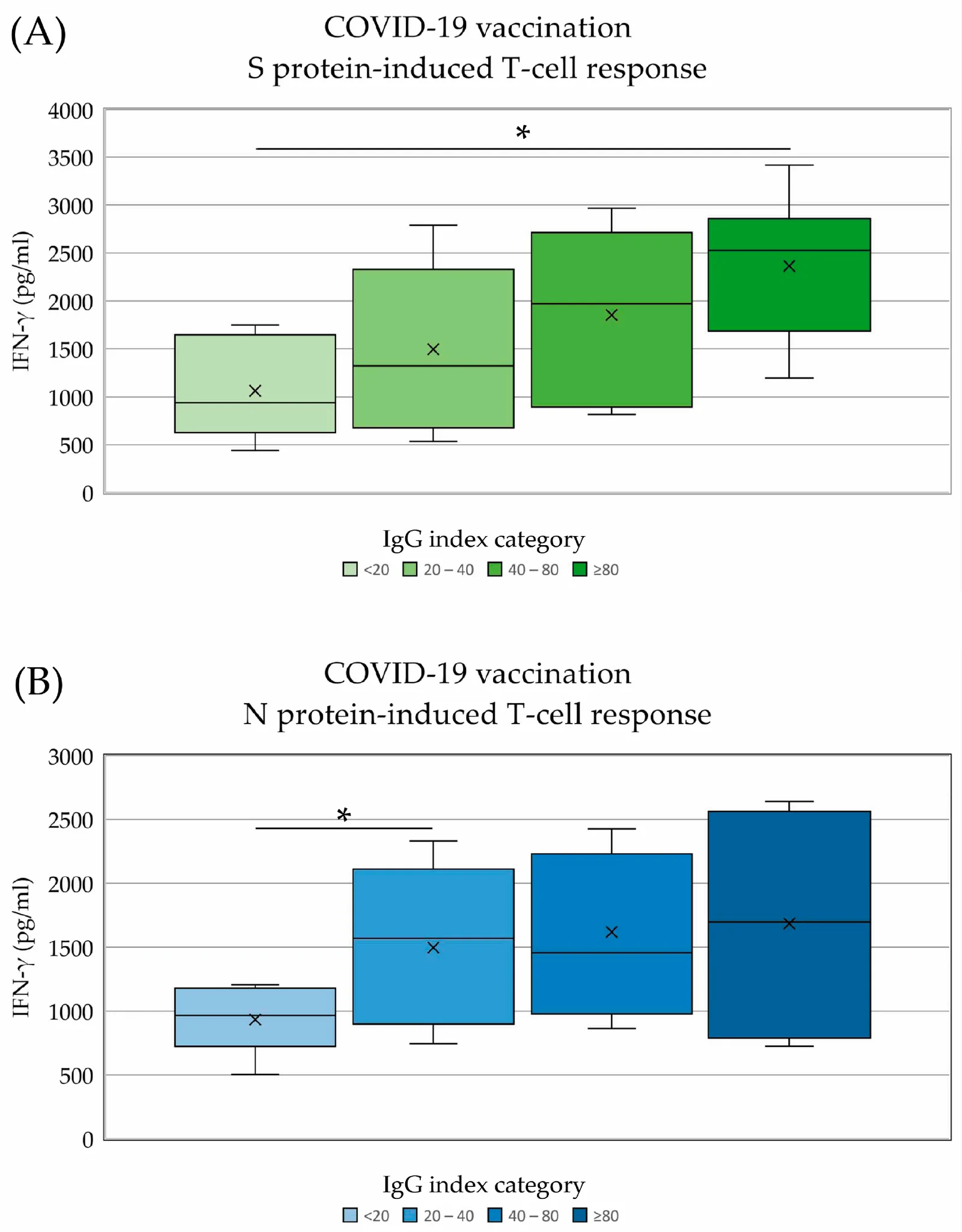
Interferon gamma (IFN-γ) release assay based on gold nanoparticles (nano-IGRA) for detection of cellular immune response to SARS-CoV-2 in subjects vaccinated against SARS-CoV-2 virus, stratified in four subgroups according to IgG antibody levels (seronegative subgroup (IgG index < 20), n = 6; low seropositive group patients (IgG index 20–40), *n* = 14; medium seropositive subgroup (IgG index 40–80), *n* = 9; and highly seropositive subgroup (IgG index ≥ 80), *n* = 6). (**A**) IFN-γ response of whole blood stimulated with SARS-CoV-2 S protein. (**B**) IFN-γ response of whole blood stimulated with SARS-CoV-2 N protein. * *p* < 0.05.

**Figure 5:**
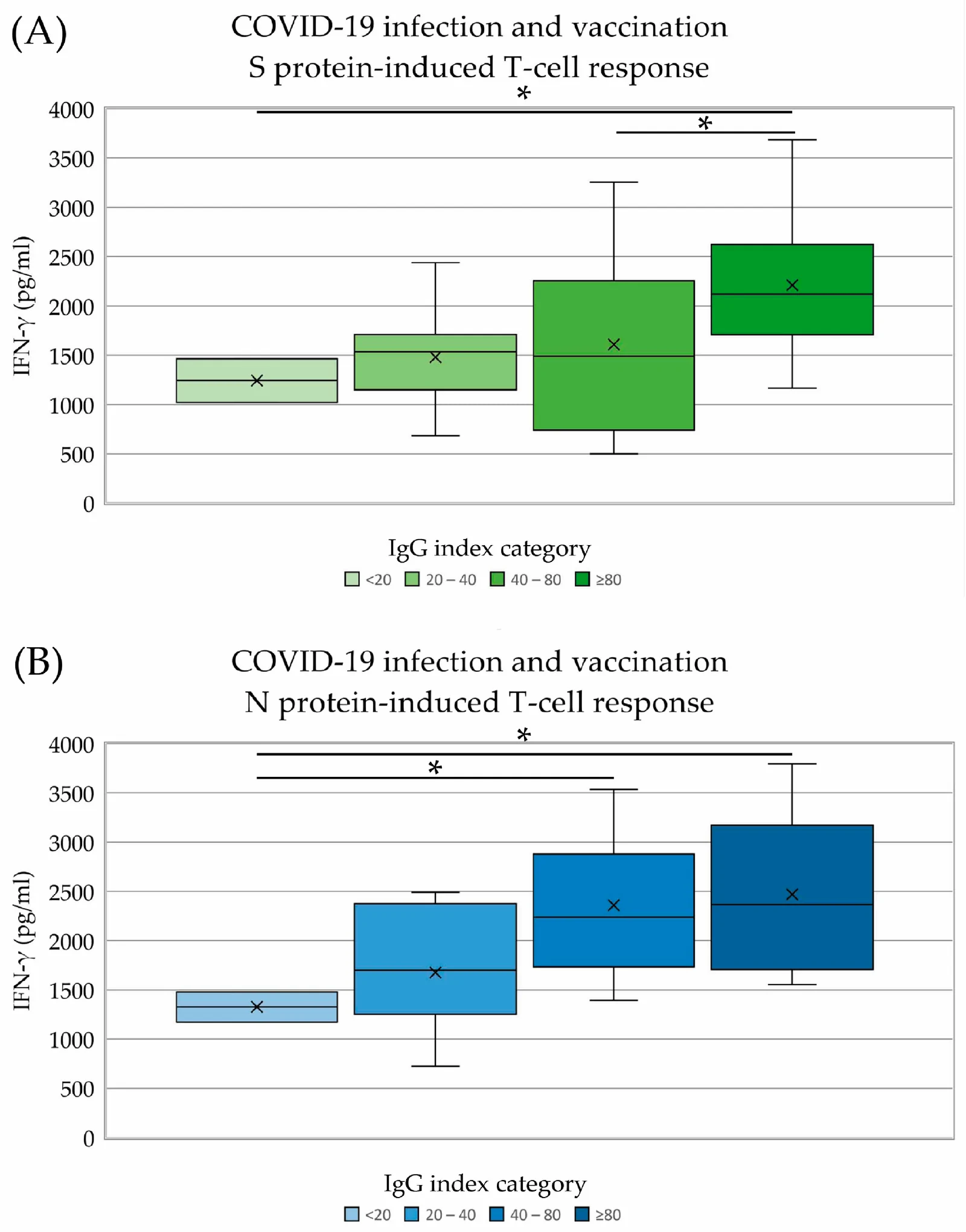
Interferon gamma (IFN-γ) release assay based on gold nanoparticles (nano-IGRA) for detection of cellular immune response to SARS-CoV-2 in subjects who had COVID-19 and who were vaccinated with SARS-CoV-2 vaccines, stratified in four subgroups according to IgG antibody levels (seronegative subgroup (IgG index < 20), *n* = 2; low seropositive group patients (IgG index 20–40), *n* = 7; medium seropositive subgroup (IgG index 40–80), *n* = 17; and highly seropositive subgroup (IgG index ≥ 80), *n* = 14). (**A**) IFN-γ response of whole blood stimulated with SARS-CoV-2 S protein. (**B**) IFN-γ response of whole blood stimulated with SARS-CoV-2 N protein. * *p* < 0.05.

**Figure 6:**
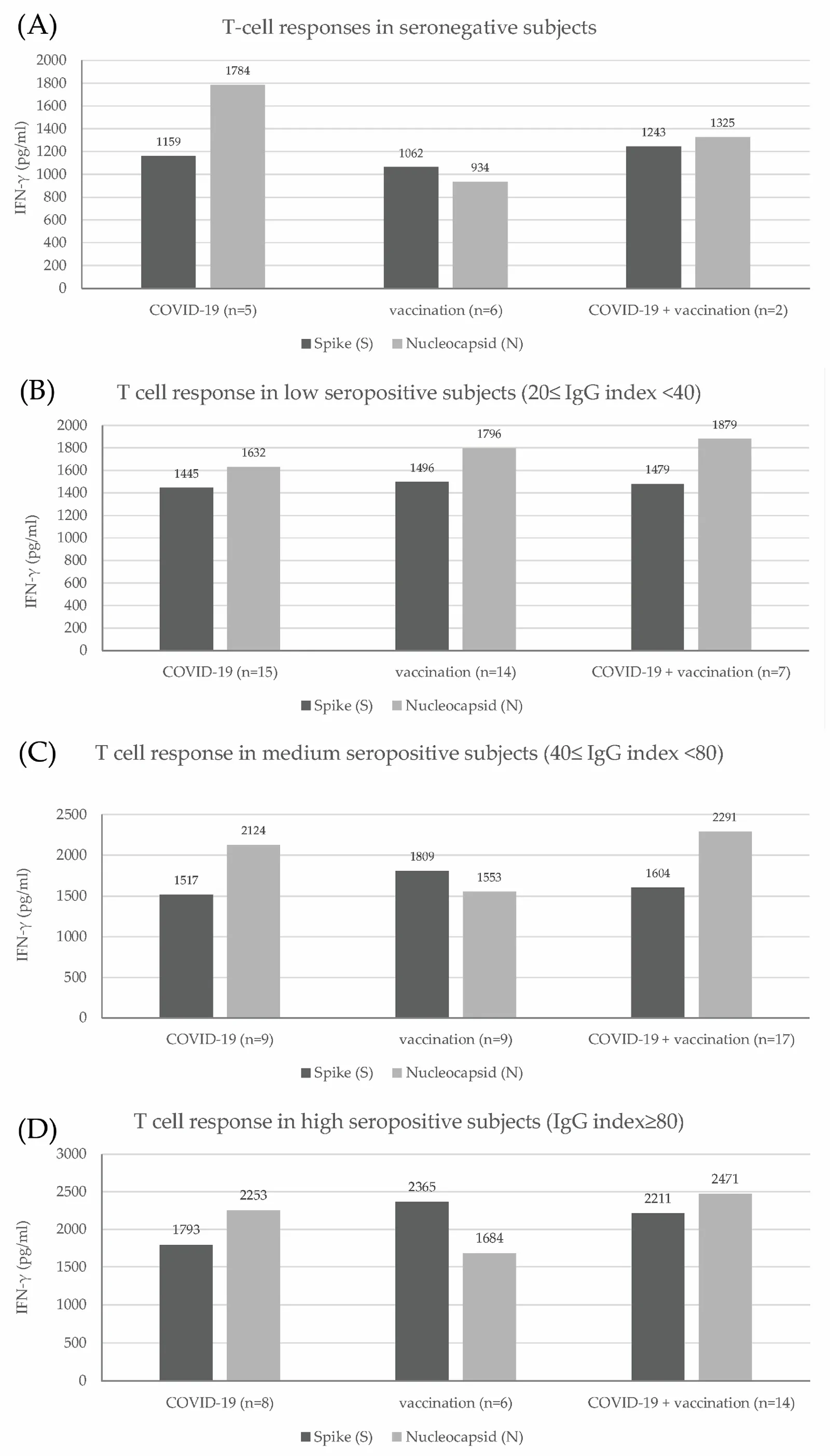
Comparison of SARS-CoV-2 Spike and Nucleocapsid protein-specific T cells responses of three groups: subjects who were only infected with COVID-19 (COVID-19 infection), subjects who were COVID-19-naïve and vaccinated with SARS-CoV-2 vaccines (vaccination) and subjects who had COVID-19 and vaccination (COVID-19 infection + vaccination). (**A**) IFN-γ level in seronegative subjects (IgG index < 20). (**B**) IFN-γ level in low seropositive subjects (20 ≤ IgG index < 40). (**C**) IFN-γ level in medium seropositive patients (40 ≤ IgG index < 80). (**D**) IFN-γ level in highly seropositive patients (IgG index ≥ 80). Bars represent the geometric means.

**Figure 7:**
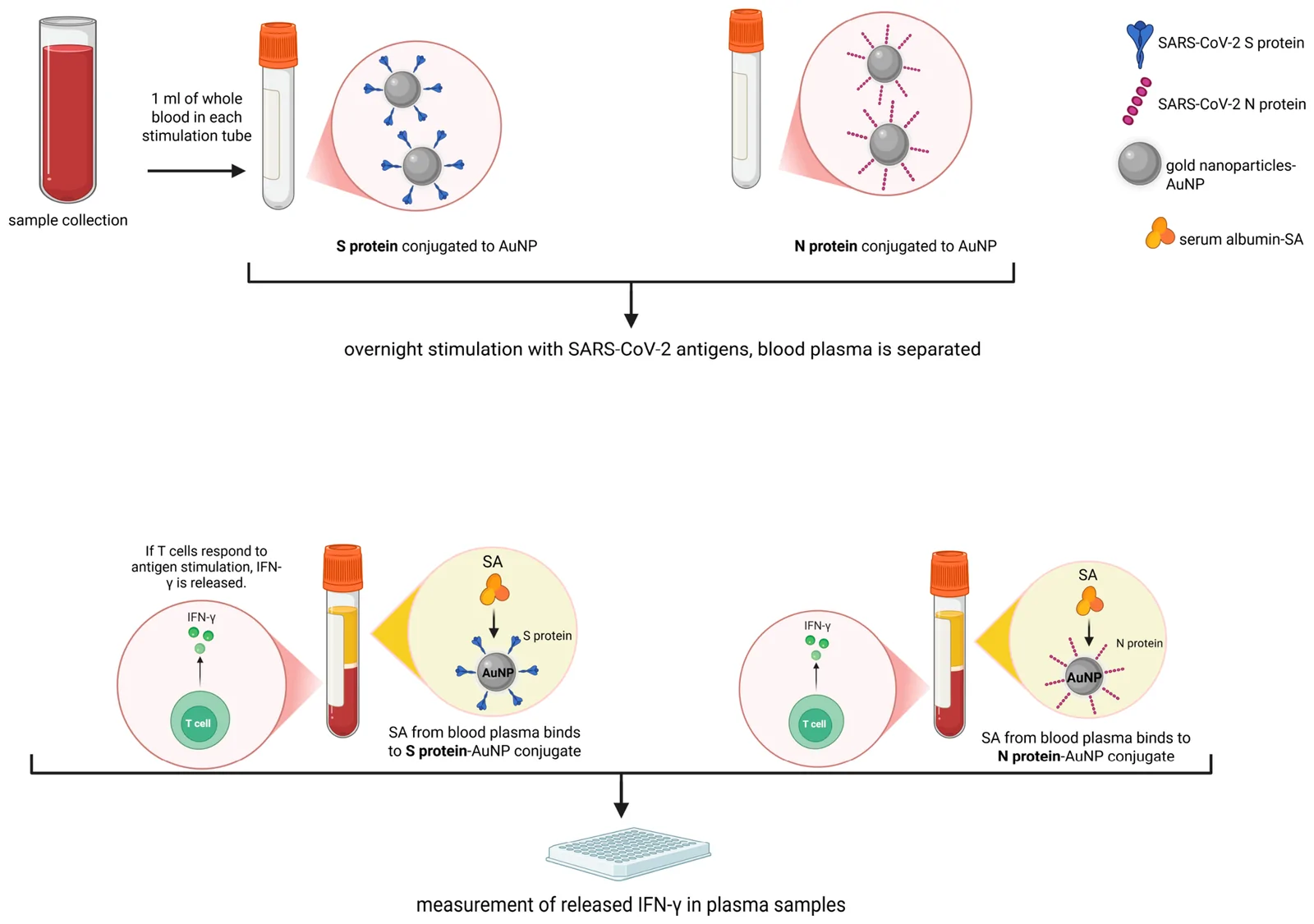
Illustration of stimulation phase in IFN-γ release assay based on gold nanoparticles (nano-IGRA). Vials coated with SARS-CoV-2 antigens conjugated to gold nanoparticles (AuNPs) are used to stimulate and analyze T cell responses. The figure was created using BioRender.com. (Created in BioRender. Todorović, A. (2026) https://BioRender.com/ctdv4zj, accessed on 9 September 2026).

**Table 1:** Demographic and clinical characteristics of participants according to SARS-CoV-2 exposure group.

Characteristic	COVID-19 Infection (*n* = 37)	Vaccination (*n* = 35)	COVID-19 + Vaccination (*n* = 40)	Total (*n* = 112)
Age, years, median (range)	42 (25–67)	39 (24–65)	44 (23–69)	42 (23–69)
female, *n* (%)	17 (46%)	18 (51%)	24 (60%)	59 (53%)
male, *n* (%)	20 (54%)	17 (49%)	16 (40%)	53 (47%)
Health status	No reported comorbidities	No reported comorbidities	No reported comorbidities	No reported comorbidities
Time since infection/vaccination to immune assessment	One month after infection	One month after first vaccine dose; no previous recorded SARS-CoV-2 infection	One month after the first vaccine dose; previous SARS-CoV-2 infection within 6 months	__
Vaccine platform	__	mRNA/viral vector/inactivated virus	mRNA/viral vector/inactivated virus	__

**Table 2:** Serostatus among three investigated groups: subjects who were only infected with COVID-19 (COVID-19 infection), subjects who were COVID-19-naïve and vaccinated with SARS-CoV-2 vaccines (vaccination) and subjects who had COVID-19 and vaccination (COVID-19 infection + vaccination).

Serostatus	IgG Index	COVID-19 Infection (*n* = 37)	Vaccination (*n* = 35)	COVID-19 Infection + Vaccination (*n* = 40)	Total (*n* = 112)
Seronegative	<20	*n* = 5 (14%)	*n* = 6 (17%)	*n* = 2 (5%)	*n* = 13 (12%)
Low seropositive	20–40	*n* = 15 (40%)	*n* = 14 (40%)	*n* = 7 (18%)	*n* = 36 (32%)
Medium seropositive	40–80	*n* = 9 (24%)	*n* = 9 (26%)	*n* = 17 (42%)	*n* = 35 (31%)
Highly seropositive	≥80	*n* = 8 (22%)	*n* = 6 (17%)	*n* = 14 (35%)	*n* = 28 (25%)

**Table 3:** Analytical evaluation and reproducibility of the nano-IGRA methodology by study group.

Group	Parameter	S Soluble	AuNP-S	*p* Value *	N Soluble	AuNP-N	*p* Value *
COVID-19 infection	Mean IFN-γ ± SD (pg/mL)	882 ± 90	1426 ± 43	*p* < 0.001	1279 ± 108	2107 ± 57	*p* < 0.001
	Intraassay CV (%)	8.3 ± 3.80	3.2 ± 1.36	*p* < 0.001	7.4 ± 3.56	3.6 ± 1.64	*p* < 0.001
	Interassay CV (%)	10.5 ± 4.43	3.5 ± 1.21	*p* < 0.001	10.1 ± 5.76	2.9 ± 1.51	*p* < 0.001
Vaccination	Mean IFN-γ ± SD (pg/mL)	992 ± 118	1613 ± 63	*p* < 0.001	872 ± 108	1637 ± 62	*p* < 0.001
	Intraassay CV (%)	7.3 ± 2.40	2.8 ± 1.25	*p* < 0.001	7.7 ± 3.12	2.7 ± 1.21	*p* < 0.001
	Interassay CV (%)	15.0 ± 9.11	3.6 ± 1.57	*p* < 0.001	15.3 ± 8.24	4.7 ± 1.82	*p* < 0.001
COVID-19 infection + vaccination	Mean IFN-γ ± SD (pg/mL)	890 ± 102	1647 ± 58	*p* < 0.001	1247 ± 101	2207 ± 76	*p* < 0.001
	Intraassay CV (%)	6.9 ± 2.95	2.5 ± 1.09	*p* < 0.001	7.0 ± 2.61	2.2 ± 1.06	*p* < 0.001
	Interassay CV (%)	12.8 ± 5.45	3.8 ± 1.26	*p* < 0.001	10.2 ± 5.83	3.7 ± 1.06	*p* < 0.001

* Statistical analysis was performed using the Wilcoxon signed-rank test. *p*-values < 0.05 were considered statistically significant.

**Table 4:** Overall analytical validation of nano-IGRA.

Assay Reproducibility	Antigen	Soluble Antigen Mean CV (%)	AuNP-Conjugated Antigen Mean CV (%)	Reduction in Variability (%)
Intraassay CV	S	7.47	2.81	62.4%
	N	7.34	2.83	61.4%
Interassay CV	S	12.77	3.62	71.7%
	N	11.86	3.76	68.3%

**Table 5:** Correlation between IgG antibody levels and IFN-γ responses measured by nano-IGRA in the three SARS-CoV-2 exposure groups.

Group	IgG vs. IFN-γ S Stimulation	IgG vs. IFN-γ N Stimulation
COVID-19 infection	ρ = 0.239; *p* = 0.153	ρ = 0.215; *p* = 0.202
Vaccination	ρ = 0.475; *p* = 0.004 *	ρ = 0.085; *p* = 0.626
COVID-19 + vaccination	ρ = 0.412; *p* = 0.0083 *	ρ = 0.371; *p* = 0.0185 *
All subjects	ρ = 0.358; *p* < 0.001 *	ρ = 0.309; *p* < 0.001 *

* Spearman’s rank correlation test was used to assess the association between IgG index values and IFN-γ concentrations. *p* < 0.05 was considered statistically significant.

**Table 6:** Comparative features and mechanistic rationale underlying the proposed AuNP-based nano-IGRA.

Feature/Aspect	Standard T-Cell Formats (e.g., LPA, ELISPOT…)	Standard IGRA Format	Nanomaterial-Enhanced IGRA (Nano-IGRA) Advantage	Nano-IGRA Rationale and Literature Support
Sample Requirement	PBMC isolation and cell culture required	Whole blood; no cell isolation	Whole blood; no cell isolation, with AuNP-conjugated antigen replacing soluble antigen	Maintains the operational simplicity of conventional IGRA while introducing a nanomaterial-based antigen presentation strategy
Primary Advantages	High sensitivity at single-cell level	Single whole-blood tube; simple workflow; low-cost; fast; scalable for clinical labs	Retains the simple whole-blood workflow, while significantly boosting signal and reproducibility	Gold nanoparticles provide a nanoscale platform for antigen presentation, which facilitate their interaction with immune cells and enhance antigen-specific cellular responses [48,49,50]
Major Inherent Limitations	Highly laborious; requires isolated PBMCs; expensive; operator-dependent; slow	High assay variability; large grey zone; signal degradation during incubation	Reduces assay variability by lowering coefficient of variation (CV%) and stabilizing readout	Proof of concept demonstrated in our study (Table 3)
Surface Area and Interaction	Limited to well-surface interaction	Soluble antigen with random cell contact	High surface area-to-volume ratio	Nanoparticles can accommodate an oriented and spatially organized antigen layer, reducing steric crowding and improving the spatial availability of the antigen for T-cell receptors (TCRs) [49,50,51,52]
Protein Matrix and Stability	Subject to rapid enzymatic cleavage	Degradation of free antigens in whole blood	Binding to serum albumin, improves matrix stability	The formation of a protein “corona” between nanoparticles and serum albumin provides a stabilizing interfacial layer; may thereby contribute to protection against premature antigen degradation during incubation [31,53,54]
Antigen Presentation Quality	Dependent on cell adherence	Monovalent binding to cell receptors	Multivalent presentation; enhanced cellular uptake and stimulation	Clustering of antigens on a nanoplatform can trigger stronger cross-linking of T-cell receptors, generating a robust IFN-γ release [49,50,52,55]
Cytokine Measurement Application	Fluorometric assay; indirect colorimetry; spot counting	Spectrophotometric readout, prone to baseline noise	Enhanced IFN-γ signal intensity with improved intra- and interassay precision	Aligns with recent advances where nanomaterial-based antigen platforms have been applied to improve cytokine-release assays in complex biological matrices such as whole blood [48,56]

## Data Availability

The original contributions presented in this study are included in the article. Further inquiries can be directed to the corresponding author.
